# The Effects of Physical Activity Together With Nutrition Programs in Educational Settings on Obesity and Overweight Reduction in Preschool Children: A Systematic Review of Randomized Controlled Trials

**DOI:** 10.1155/jnme/9563746

**Published:** 2026-01-22

**Authors:** Markel Rico-González, Adrián Moreno-Villanueva, Carlos D. Gómez-Carmona, Jorge Carlos-Vivas

**Affiliations:** ^1^ Department of Didactics of Music, Plastic and Body Expression, University of the Basque Country, UPV-EHU, Leioa, Spain; ^2^ Faculty of Health Science, University Isabel I, Burgos, 09003, Spain; ^3^ Department of Music, Plastic and Body Expression, Research Group in Training, Physical Activity and Sports Performance (ENFYRED), University of Zaragoza, Teruel, 44003, Spain, unizar.es; ^4^ Faculty of Sport Sciences, Physical Activity for Education, Performance and Health (PAEPH) Research Group, University of Extremadura, Cáceres, 10003, Spain, unex.es

**Keywords:** body composition, bone health, micronutrients, motor performance, school-based intervention, synergistic effects

## Abstract

The first years of life are crucial to start preventive interventions that can have an impact on lifestyle and later overweight and obesity. Being obese during preschool years increases the likelihood of remaining obese as an adult and is associated with serious health conditions. Combined physical activity and nutritional interventions may produce synergistic effects on child development, but evidence from school‐based programs is still limited. This systematic review investigated the impact of physical activity programs with simultaneous nutrition‐related interventions in obese preschoolers. Methods: Systematic search across five databases (PubMed, ProQuest, SCOPUS, Web of Science, and SPORTDiscus) up to August 31, 2025. Randomized controlled trials examining combined in‐preschool physical activity and nutrition‐related interventions in preschool children recruited from educational settings were included. Methodological quality was rated using the RoB‐2 scale. This systematic review was registered in PROSPERO. Conclusions: Combined preschool‐based interventions integrating physical activity and nutrition show modest but consistent benefits in reducing BMI and improving dietary and behavioral outcomes in obese preschoolers. Family involvement and structural changes in the school environment appear to enhance effectiveness. Despite methodological limitations and heterogeneity across trials, evidence supports preschool years as a critical window for obesity prevention. Future studies should adopt standardized outcomes, longer follow‐up, and cost‐effectiveness analyses to inform large‐scale implementation.

## 1. Introduction

One of the most significant public health challenges of the twenty‐first century is childhood obesity. Prevalence rates have increased in developed and developing nations over the past 4 decades [1, 2]. The World Health Organization estimates that over 390 million children and adolescents were overweight in 2022, including 160 million living with obesity [3]. The global prevalence of obesity among children aged 5–19 years has increased more than 10‐fold since 1975, and recent forecasting studies project that by 2050, approximately 15.6% of children aged 5–14 years globally will have obesity [4]. Children who were obese in preschool years are more likely to remain obese throughout adolescence and into adulthood. Evidence demonstrated that approximately 55% of obese children go on to be obese in adolescence, and around 80% of obese adolescents will still be obese in adulthood [5, 6]. Also, childhood obesity is associated with serious health conditions including type 2 diabetes, cardiovascular disease, metabolic syndrome, and psychosocial difficulties [7, 8]. The economic burden is also substantial, with projected global costs of overweight and obesity reaching US$3 trillion per year by 2030 [3].

The preschool period comprised ages from 2 to 6 years. It represents a critical developmental window characterized by rapid physical growth, cognitive development, and the establishment of health behaviors that tend to persist into adolescence and adulthood [9, 10]. During these years, children develop food preferences, eating behaviors, and activity patterns that are strongly influenced by their immediate environment (e.g., family, childcare settings, and community factors) [11]. Research indicates that dietary habits and physical activity levels established during early childhood significantly predict behavioral patterns in adolescence and adulthood. Previous evidence shows that combinations of healthy or unhealthy lifestyle behaviors can persist from as early as 1.5 years of age [12]. The neuroplasticity and behavioral malleability characteristic of this developmental stage make preschool children particularly responsive to obesity prevention interventions [13]. Early childhood obesity is particularly difficult to reverse, with 72% of children under 5 with obesity experiencing obesity in adolescence [14]. Furthermore, the anthropometric characteristics and adherence to physical activity guidelines during preschool years significantly influence children’s physical fitness, motor skills development, and overall health trajectories [15, 16].

Educational settings have been identified as ideal spaces to apply obesity prevention programs due to their reach, existing infrastructure, and the substantial amount of time children spend in these environments [17, 18]. Children attending full‐time preschool programs typically consume one‐to‐two meals at these facilities, representing a significant proportion of their total daily energy intake. Multicomponent interventions that combine nutrition education, physical activity promotion, and environmental modifications within educational settings have shown promise in preventing excessive weight gain [19, 20]. Systematic reviews and meta‐analyses have demonstrated that comprehensive programs addressing both dietary intake and physical activity behaviors can produce meaningful improvements in body mass index (BMI), cardiometabolic biomarkers, dietary quality, and physical activity levels among preschool‐aged children [21–23]. The 2019 Cochrane review of childhood obesity prevention interventions found that diet combined with physical activity interventions can reduce the risk of obesity in young children aged 0–5 years [24]. Recent evidence also indicates that physical activity programs combined with nutritional supplementation can effectively improve children’s cardiometabolic health and nutritional status when implemented in educational settings [25, 26].

Despite growing recognition of the importance of early intervention, the effectiveness of combined physical activity and nutrition programs in educational settings for preschool obesity prevention remains inconsistent [27]. This variability may be attributed to differences in intervention design, including the presence or absence of family involvement components, the duration and intensity of interventions, the training and engagement of preschool staff, and the socioeconomic and cultural contexts of participating families [28]. Understanding which intervention components are most effective, for which populations, and under what circumstances is essential for translating research findings into scalable, sustainable prevention programs. Cost‐effectiveness evaluations have shown that many interventions offer value for money, with childcare center‐based programs demonstrating potential lifetime medical cost savings, as obese children spend approximately $19,000 more on medical care compared to normal‐weight children [29].

Given the urgent need for effective obesity prevention strategies during the preschool years and the current gaps in the evidence base, a comprehensive synthesis of randomized controlled trials examining combined physical activity and nutrition interventions in educational settings is warranted. Therefore, this systematic review aims to evaluate the effectiveness of multicomponent programs implemented in preschool and childcare settings on anthropometric outcomes, dietary behaviors, and physical activity levels among children aged 2–6 years. By synthesizing evidence, this review seeks to identify effective intervention components, optimal program characteristics, and factors that moderate intervention effectiveness. The findings will provide valuable guidance for researchers, policymakers, educators, and public health practitioners working to develop and implement evidence‐based obesity prevention programs for young children.

## 2. Methods

### 2.1. Experimental Approach to the Problem

The present systematic review was conducted in accordance with the Preferred Reporting Items for Systematic Reviews and Meta‐Analyses (PRISMA) guidelines [30] and adhered to established guidelines for conducting systematic reviews within the domain of sports sciences [31]. The review protocol was developed with the objective of ensuring comprehensive coverage of the relevant literature while maintaining methodological rigor. This systematic review was registered in PROSPERO: CRD420251265996 (provisional–pending acceptance).

### 2.2. Information Sources

The following bibliographic databases were consulted: Web of Science, PubMed, SPORTDiscus, ProQuest Central, and SCOPUS. The search encompassed all published literature before August 31, 2025. The combination of databases was selected to ensure broad coverage of both medical and sports science literature.

### 2.3. Data Collection

The PICO (Patient, Problem, or Population–Intervention or Exposure–Comparison, Control, or Comparator–Outcome[s]) framework was implemented to structure the search strategy and ensure systematic coverage of relevant literature. In the interest of maintaining transparency, the authors were not blinded to journal names or manuscript authors. The final search string was as follows:

(“early childhood” OR preschool OR kindergarten) AND (obese OR overweight) AND (Nutrient^∗^ OR nutrition OR food^∗^ OR diet^∗^) AND (exercise OR “Physical activity” OR “physical education” OR sport OR fitness OR aerobic OR movement) AND (program^∗^ OR intervention) AND (“randomized controlled trial^∗^”)

### 2.4. Eligibility Criteria

The authors initiated the search string on databases and downloaded the title, authors’ names, journal, and date of all the articles that appeared in the search. Following the organization of the Excel spreadsheet, the process of removing all duplicates was initiated, and the remaining articles were subjected to a rigorous evaluation to ascertain their eligibility. The specific inclusion and exclusion criteria used for study selection are detailed in Table 1.

**Table 1:** Inclusion and exclusion criteria.

Item	Inclusion	Exclusion	Search coherence
Population	Preschool children	Children out of preschool or school age Children under medical treatment	*(“early childhood” OR preschool OR kindergarten OR* *s* *c* *h* *o* *o* *l* ^∗^ *OR “primary education” OR “elementary education”) AND (obese OR overweight)*

Intervention or Exposure	Studies that presented and intervention aimed to prevent obesity. In this program, children must practice, exercise during preschool hours (in kindergartens or childcare centers) as part of a multifaceted intervention program focusing on nutrition and exercise	Studies not mainly aimed at presenting an intervention to prevent obesity Children out of school doing physical activity program (nutrition intervention could be at home) Interventions target parents Home‐based or community‐based interventions Children not involved in a multifaceted program Children involved in programs with supplementation (e.g., vitamins) Study protocols	*(* *n* *u* *t* *r* *i* *e* *n* *t* ^∗^ *OR nutrition OR* *f* *o* *o* *d* ^∗^ *OR* *D* *I* *E* *T* ^∗^ *) AND (exercise OR “Physical activity” OR “physical education” OR sport OR fitness OR aerobic OR movement) AND (* *p* *r* *o* *g* *r* *a* *m* ^∗^ *OR intervention)*

Comparation	—	—	—

Outcome(s)	Any outcome	—	

Design	Randomized controlled trial	Nonrandomized controlled trials	*“randomized controlled* *t* *r* *i* *a* *l* ^∗^ *”*

Other criteria	Peer‐reviewed full‐text studies published in original journal articles Studies written in English or Spanish	Non‐peer‐reviewed journal articles Nonoriginal full‐text studies (conference papers…) Studies written in other language	

### 2.5. Data Extraction

A standardized data extraction process was implemented using an Excel spreadsheet developed in accordance with the Cochrane Consumers and Communication Review Group’s data extraction template. The spreadsheet enabled a systematic evaluation of the inclusion and exclusion requirements for all the selected studies. The extraction process was conducted independently by two authors, with any disagreements being resolved through discussion until consensus was reached. A full record was kept of all articles that were not included, including the particular reasons for exclusion. The data were systematically recorded and stored in a spreadsheet.

### 2.6. Assessment of Study Methodology

The second version of the Cochrane risk‐of‐bias tool for cluster randomized trials (RoB 2 CRT) was used. The authors assessed different key domains: randomization process (Domain 1a), timing of recruitment (Domain 1b), deviations from intended interventions (Domain 2), missing outcome data (Domain 3), measurement of the outcome (Domain 4), and selection of the reported result (Domain 5) [32]. Each domain was evaluated and classified using low risk of bias (+), high risk of bias (−), or some concerns or unclear risk of bias (?). Two authors conducted RoB 2 CRT assessments, with disagreements resolved through discussion or consultation with a third author.

## 3. Results

### 3.1. Study Selection and Inclusion Process

**Figure 1:**
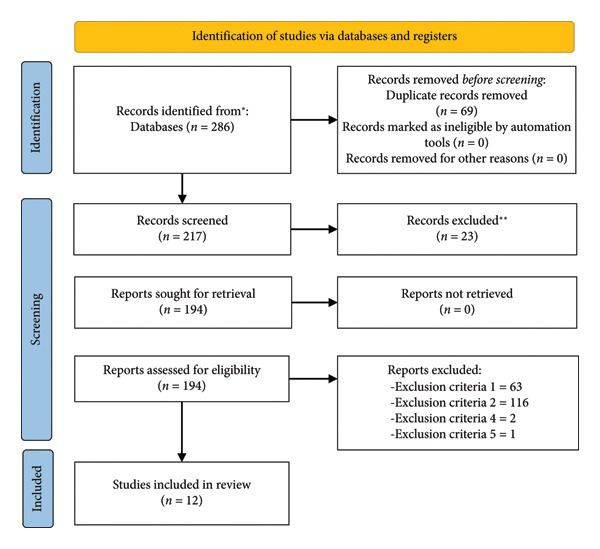
PRISMA flow diagram.

After analyzing all databases (WoS: 26; PubMed: 33; SPORTDiscus: 4; ProQuest Central: 8; and SCOPUS: 215), the contents of 286 articles were checked, detecting, at the initial stage, 69 duplicate articles. Then, the authors analyzed if each of the remaining 217 articles met all inclusion criteria, resulting in the elimination of 317 articles by exclusion criteria number one (*n* = 63), exclusion criteria number two (*n* = 116), exclusion criteria number four (*n* = 2), exclusion criteria number five (*n* = 1), and exclusion criteria number six (*n* = 23). The remaining 12 articles were included in the qualitative synthesis of the systematic review (Figure 1).

### 3.2. Methodological Quality

The methodological quality assessment, performed using the RoB 2 CRT, revealed pervasive methodological weaknesses across the included studies. All the trials (12/12) were judged to be at an overall high risk of bias. This critical judgment was systematically determined by significant concerns in at least one domain for every study, with the most decisive factor being Domain 2 (Bias due to Deviations from Intended Interventions). The intricate and behavioral character of the interventions in all the included trials rendered effective blinding of participants and intervention personnel unfeasible, thus introducing an almost unavoidable, high risk of performance bias. It was consistently noted that there were additional substantial weaknesses across several domains. Domain 5 (Bias in Selection of the Reported Result) was identified as a potential area for improvement, with several studies prioritizing and emphasizing significant exploration or subgroup analyses following nonsignificant primary outcomes. Domain 1b (Timing of Recruitment) was also identified as a point for consideration, due to the enrollment of individual participants after the clusters had been randomized. Finally, Domain 3 (Missing Outcome Data) raised significant concerns about bias arising from loss to follow‐up, with attrition rates (in several trials exceeding 25%) being a particular point of concern. In most trials, the predicted direction of the identified bias consistently favored the magnitude of the effect of the experimental intervention. The results of the risk‐of‐bias assessment are presented in Table 2.

**Table 2:** Risk of bias using RoB 2 CRT.



*Note:* : high risk, : low risk, : some concerns.

### 3.3. Study Characteristics

#### 3.3.1. Quantitative Synthesis

The effectiveness of multifaceted interventions in preventing and managing obesity among preschool children varied across the 12 studies reviewed. The quantitative synthesis of anthropometric outcomes showed mixed results. While several studies, including Davis et al. [33], Rhee et al. [34], Nyberg et al. [38], Ling et al. [40], and Jouret et al. [42], found no statistically significant difference in BMI z‐score trajectories between intervention and control groups, other interventions reported favorable outcomes. For instance, Gato‐Moreno et al. [35] observed a significant reduction in BMI z‐scores in the intervention group (−0.14 units) compared to an increase in the control group (+0.12 units). Similarly, Natale et al. [36, 41] reported a significant decrease in BMI percentile, which was sustained at a 2‐year follow‐up. Zask et al. [43] reported a significant difference in mean BMI z‐score change (−0.18 intervention vs. −0.03 control). Niederer et al. [39] reported a beneficial effect on body fat and waist circumference, and Fitzgibbon et al. [44] demonstrated that their “Hip‐Hop to Health Jr.” intervention resulted in a smaller increase in BMI over 2 years compared to controls among minority preschoolers. Reyes‐Morales et al. [37] also reported positive changes in adiposity indicators. Overall, seven studies showed significant reductions in BMI or BMI z‐scores, reflecting a positive impact of the interventions in a little more than half of the included trials.

Beyond anthropometric measures, nine studies reported positive effects on at least one obesity‐related behavioral outcome. Rhee et al. [34] observed improved inhibitory control in younger children, while Reyes‐Morales et al. [37] and Niederer et al. [39] reported positive changes in physical activity. Ling et al. [40] observed reductions in body fat percentage and increased fruit and vegetable intake, and Zask et al. [43] reported improvements in movement skills and dietary intake. Changes in dietary behaviors were predominantly evaluated using parent‐reported tools. Validated Food Frequency Questionnaires (FFQ) were utilized in studies, such as Davis et al. [33], Nyberg et al. [38], and Niederer et al. [39] to assess the frequency of fruit and vegetable consumption. Other studies, such as Fitzgibbon et al. [44], employed 24‐h dietary recalls to estimate caloric intake and macronutrient composition. Adherence was typically measured by changes in daily servings of fruits, vegetables, and sugar‐sweetened beverages. Zask et al. [43] also used observation/checklists (e.g., lunchbox audits and staff observation) to monitor dietary practices in the preschool setting.

#### 3.3.2. Qualitative Synthesis

The interventions employed a variety of strategies, such as nutrition education, physical activity promotion, encouraging parental involvement, making environmental modifications, and introducing policy changes. Most programs were implemented in childcare or preschool settings and targeted children from low‐income, multiethnic, or disadvantaged communities. Several studies, such as those by Gato‐Moreno et al. [35], Natale et al. [36, 41], and Fitzgibbon et al. [44], emphasized the importance of parental education and engagement in achieving favorable outcomes. However, others, such as Davis et al. [33] and Nyberg et al. [38], highlighted the challenges of implementing long‐term, community‐wide interventions with limited impact on BMI. Cognitive‐behavioral approaches, as demonstrated by Rhee et al. [34], showed potential in modifying eating behaviors, though not in reducing calorie intake. Interventions tailored to specific sociocultural contexts, such as those by Jouret et al. [42] and Niederer et al. [39], demonstrated effectiveness in reducing overweight prevalence and improving fitness. Overall, the findings suggest that multifaceted, context‐sensitive, and sustained interventions involving families and educators are more likely to produce meaningful changes in obesity‐related outcomes among preschool‐aged children.

The characteristics of studies were extracted and are clustered into Table 3.

**Table 3:** Main characteristics and findings about the effects of multifaceted intervention programs aimed at preventing and managing preschoolers’ obesity.

Authors	Sample characteristics	Multifaceted intervention program	Outcome measures	Main results	Conclusions and applications
Davis et al. [33]	1816 preschool children aged 2–5 years old, from predominantly Hispanic and American Indian communities, who attend to Head Start centers in rural areas (33% obese or overweight at baseline)	‐ Duration: 5 years ‐ Components: 1. Classroom Curriculum: nutrition and physical activity, 30 min of physical activity daily 2. Quarterly professional development training for teachers and food service staff 3. Policy and behavior change in food purchasing and preparation by food service staff 4. Family Component: take‐home materials and family events twice during the school year 5. Local Grocery Store Component: increasing availability and visibility of healthier food options, providing recipes and nutrition information 6. Healthcare Provider Component: emphasizing healthy eating and physical activity during patient visits, attending CHILE family events	‐ BMI *z*‐score	‐ No significant difference in BMI *z*‐scores between the intervention and comparison groups	‐ A 5‐year intervention to prevent obesity in Hispanic and American Indian preschool children in rural New Mexico did not show significant differences in BMI z‐scores between groups after 2 years ‐ Obesity prevention in Hispanic and American Indian preschool children in rural communities is challenging and complex ‐ Changes in obesity rates may require more than 2 years to achieve

Rhee et al. [34]	91 preschool children aged 4–6 years old, racially and ethnically diverse (BMI ≥ 5th percentile)	‐ Duration: Three weeks ‐ Frequency: Three times a week ‐ Duration Per Session: 30 min ‐ Components: 1. Dramatic/pretend play to exercise inhibitory control, working memory, and cognitive flexibility 2. Visual and verbal scaffolding to support and remind children of their goals or behaviors 3. Games to help children work on increasing working memory and inhibitory control 4. Self‐talk to remind children of their goal behaviors. ‐ Goals: Educate children about energy‐dense snack foods and train them to inhibit responses to these foods	‐ Calories consumed during the postintervention “Eating in the Absence of Hunger” paradigm, inhibitory control assessed using “Day/Night” and “Less is More” tasks	‐ No significant reduction in overall calorie consumption between groups ‐ Overweight/obese children in the intervention group consumed similar calories to healthy‐weight children, unlike in the control group ‐ Younger children in the intervention group showed improved inhibitory control compared to their counterparts in the control group	A pilot program to enhance self‐regulation in preschool children did not decrease overall energy‐dense food consumption but may help temper excess calorie intake in overweight/obese children

Gato‐Moreno et al. [35]	261 preschoolers aged 3–4 years old	‐ Duration: 2 years ‐ Initial Intervention (12 Weeks): Six 2‐h group training sessions, given every 2 weeks, covering topics, such as introduction to nutrition, healthy eating habits, menu planning, physical activity, and food labeling ‐ Follow‐Up Intervention (1 Session): One 3‐h session 1 year later, reviewing nutrients, healthy menus, and physical activity ∗ Specific interventions are described in Table 3​ of the analyzed manuscript	‐ BMI *z*‐score	‐ The intervention significantly decreased the zBMI in the intervention group after the first year and the subgroup with higher initial zBMI ‐ The joint prevalence of overweight and obesity increased in the control group but remained stable in the intervention group ‐ The educational intervention was effective in improving children’s BMI and preventing overweight and obesity, especially in those with higher initial BMI values	Early nutritional education intervention with parents improved children’s BMI and prevented overweight/obesity

Natale et al. [36]	307 multiethnic normal weight at baseline children aged 2–5 years old	‐ Healthy Inside–Healthy Outside (HI‐HO) program ‐ Duration: 6 months ‐ Components: 1. Intervention centers received healthy menu changes 2. Family‐Based Education Focused on: increased physical activity, fresh produce intake, decreased intake of simple carbohydrate snacks, and decreased screen time	‐ BMI *z*‐score ‐ Nutrition practices (consumption of junk food, fresh fruits and vegetables, juice, and milk)	‐ The intervention significantly reduced BMI *z*‐scores and improved nutrition practices among preschool‐aged children ‐ Children in the intervention group consumed less junk food and more healthy food compared to the control groups ‐ 97 percent of normal‐weight children at baseline remained normal weight 12 months later, supporting the program’s effectiveness	A childcare center‐based obesity prevention program reduced BMI z‐scores and improved nutrition practices among preschool‐aged children

Reyes‐Morales et al. [37]	674 children aged 2–4 years old, from childcare centers	‐ Duration: 12 months ‐ Components: 1. Kindergarten Staff Training: 3‐day (24‐h) interactive workshop for educators and childcare staff; focusing on child nutrition and physical activity behaviors, daycare environment, and parent engagement strategies 2. Child Education Sessions (Once a Week): 12‐weekly interactive educational sessions combining nutrition and physical activity. Activities included taste testing healthy snacks, choosing healthier options, and culturally adapted games. Sessions supported by a tailored implementation manual provided to each daycare center 3. Family Workshops (Once Every 2 Months): Six bimonthly participatory workshops aligned with child session content. They included key messages to support behavior change at home, reinforcement tools included monthly goal posters and game/message cards for ongoing parent engagement	‐ Changes in dietary habits ‐ Changes in physical activity ‐ Food availability at home ‐ Maternal feeding styles	‐ The intervention resulted in a decrease in the availability of nonrecommended foods at home and an increase in physical activity in the intervention group ‐ Improvement in physical activity is noted as potentially effective in the long term ‐ There is a need for innovative strategies to modify family dietary risk behaviors	A 12‐month multifaceted intervention based on childcare centers reduced availability of unhealthy foods at home and increased physical activity in preschool children

Nyberg et al. [38]	378 six‐year‐old children and their parents from disadvantaged areas of Stockholm	‐ Duration: 6 months ‐ Components: 1. Health Information for Parents: a brochure sent home to parents, aiming to increase knowledge on promoting healthy dietary and physical activity habits 2. Motivational Interviewing with Parents: two individual sessions, approximately 45 min each, targeting parental self‐efficacy and willingness to change 3. Teacher‐Led Classroom Activities with Children: 10 30‐min sessions, aiming to increase children’s knowledge and influence attitudes toward healthy behaviors	‐ Consumption of unhealthy foods ‐ Consumption of unhealthy drinks ‐ Physical activity ‐ BMI standard deviation score	‐ The intervention significantly reduced consumption of unhealthy foods and drinks among children ‐ The effect on unhealthy food intake was sustained for boys at follow‐up ‐ There was no significant effect on physical activity, and the positive effect on weight development among obese children was not sustained	A universal parental support program can promote healthy dietary behaviors and prevent obesity in disadvantaged 6‐year‐old children but had no impact on physical activity and only transient effects on weight development for obese children, indicating a need for prolongation or intensification for sustainable effects

Niederer et al. [39]	652 preschool children from areas with a high migrant population from two different sociocultural and linguistic regions in Switzerland	‐ Duration: One school year ‐ Components: 1. Physical Activity Program: Four 45‐min sessions per week, with initial health promoter involvement reduced to twice a month after 4 months, and remaining sessions led by regular preschool teachers 2. Nutrition Lessons: 22 lessons based on Swiss Society of Nutrition recommendations 3. Media Use and Sleep Lessons: included in the 22 lessons 4. Environmental Adaptations: installation of fixed and mobile equipment to promote PA	‐ BMI ‐ Aerobic fitness (multistage 20 m shuttle run test)	‐ The intervention had a more beneficial effect on waist circumference in overweight children compared to normal‐weight children ‐ The intervention had more beneficial effects on BMI, sum of four skinfolds, and waist circumference in low‐fit children compared to normal‐fit children ‐ The effects on fitness outcomes were not significantly different between overweight and normal‐weight children or between low‐fit and normal‐fit children	A multidimensional school‐based lifestyle intervention reduced adiposity and improved fitness in overweight or low‐fit preschoolers

Ling et al. [40]	130 preschoolers (mean age: 49.27 months) from low‐income backgrounds	‐ “FirstStep2Health” Intervention: daycare‐based, multicomponent intervention targeting preschoolers and parents/legal guardians ‐ Duration: 10 weeks ‐ Components: 1. Participatory learning for children 2. Weekly habit‐formation tasks for parents on child feeding practices and physical activity	‐ MVPA (minutes/hour) ‐ Dietary intake (24‐h recall). ‐ Skin carotenoids (fruits/vegetables intake) ‐ BMI ‐ Body fat percentage	‐ The FirstStep2Health intervention significantly reduced total fat intake and percentage body fat in preschoolers ‐ There was a significant increase in fruit and vegetable intake as measured by skin carotenoids ‐ The intervention had medium effects on reducing anthropometric measures, such as BMI, BMI percentile, and BMI *z*‐score	The FirstStep2Health intervention showed promising effects in reducing obesity‐related anthropometric measures among low‐income preschoolers, although it did not improve physical activity levels, indicating potential in obesity prevention despite some limitations

Natale et al. [41]	1211 multiethnic children from low‐income families	‐ Duration: 2 years ‐ Components: 1. Menu modifications 2. Healthy eating and physical activity curriculum for children 3. Parent curriculum for healthy meal preparation, reinforced through a role‐modeling curriculum	‐ BMI percentile ‐ Dietary patterns (consumption of fruits/vegetables and unhealthy food)	‐ Children in the intervention group had a less significant increase in BMI percentile compared to the control group ‐ Obese children in the intervention group showed a significant increase in fruit and vegetable consumption compared to the control group obese children ‐ The intervention maintained healthy BMI percentile levels over 2 years among low‐income, multiethnic children	The “Healthy Caregivers–Healthy Children” intervention resulted in the maintenance of healthy body mass index percentile over two preschool years among low‐income multiethnic children by reducing the increase in BMI percentiles and increasing fruit and vegetable consumption among obese children

Jouret et al. [42]	1780 preschool children aged 3–4 years old, differed by age and socioeconomic status (underprivileged vs. nonunderprivileged)	‐ Duration: 2 years. ‐ Components Per Group: 1. EPIPOI‐1: parents and teachers received basic information on overweight and health; children underwent screening for overweight or risk of overweight; follow‐up care by physicians 2. EPIPOI‐2: includes all components of EPIPOI‐1 plus kindergarten‐based education to promote healthy practices related to nutrition, physical activity, and sedentary behaviors	‐ Prevalence of overweight ‐ BMI *z*‐score ‐ Change in BMI *z*‐score	‐ In underprivileged areas, both intervention groups had significantly lower prevalence of overweight and BMI *z*‐scores compared to controls after the intervention ‐ EPIPOI‐1 showed a significant effect on reducing overweight prevalence in underprivileged areas ‐ In nonunderprivileged areas, the EPIPOI‐2 group had a lower gain in BMI *z*‐score compared to controls and EPIPOI‐1	Kindergarten‐based interventions, including screening and education, can reduce overweight prevalence in preschool children, especially in underprivileged areas

Zask et al. [43]	560 preschool children aged 3–6 years old	‐ “Tooty Fruity Vegie” intervention ‐ Duration: 10 months ‐ Components: Nutrition and physical activity strategies 1. Policy and Environmental Changes: review and update of food/nutrition policies to define suitable lunchbox items, playground modifications to promote active play, improved access to drinking water, and provision of small grants for sports equipment 2. Parent Engagement and Education: communication of new lunchbox policy with visual displays, distribution of educational DVD modeling healthy eating practices, workshops on positive parenting and managing fussy eaters, workshop on reducing sedentary behavior and promoting physical activity, and monthly newsletter with tips on healthy eating and active play 3. Child‐Focused Activities: use of puppets, costumes, storytelling, role‐play, gardening, cooking, and taste testing to teach food concepts, consistent messaging about “everyday” versus “sometimes” foods, and staff modeling healthy behaviors and providing positive reinforcement 4. Physical Activity Promotion: structured fundamental movement skill sessions twice weekly, and enhanced access to sports equipment during free play	‐ Fundamental movement skills ‐ Fruit and vegetable intake ‐ Unhealthy food items ‐ Waist circumference growth ‐ BMI *z*‐scores	‐ Children in intervention preschools significantly improved movement skills and had more fruit and vegetable serves, with fewer unhealthy food items in their lunch boxes ‐ There was a significant reduction in waist circumference growth and BMI *z*‐scores ‐ The intervention produced significant changes in children’s food intake, movement skills, and indicators of weight status	The 10‐month “Tooty Fruity Vegie” preschool intervention improved children’s movement skills, fruit/vegetable intake, and weight status

Fitzgibbon et al. [44]	412 preschool children aged 3–5 years old at risk of overweight, from minority ethnic groups and low‐income background	‐ “Hip‐Hop to Health Jr.” Intervention: A 14‐week obesity prevention program for minority preschool children ‐ Duration: September 1999 to June 2002 ‐ Components: 1. Weekly lessons, including food group puppets, interactive songs, and aerobic activity, to teach healthy eating and exercise habits 2. CD with music and exercise routines. 3. Weekly parent newsletters with homework assignments to reinforce learning at home	‐ BMI	‐ Intervention children had significantly smaller increases in BMI compared to control children at both 1‐year and 2‐year follow‐ups ‐ The intervention significantly reduced the percentage of calories from saturated fat at the 1‐year follow‐up ‐ The “Hip‐Hop to Health Jr.” intervention is effective in reducing subsequent increases in BMI in preschool children, particularly among minority children	The culturally proficient dietary/physical activity “Hip‐Hop to Health Jr.” intervention was effective in reducing increases in BMI in preschool minority children over a 2‐year period, indicating a promising approach to prevention of overweight in this demographic

Abbreviations: BMI, body mass index; MVPF, moderate–vigorous physical activity.

## 4. Discussion

This review confirms that preschool‐based multifactorial interventions (nutrition + physical activity) have the potential to yield positive anthropometric and behavioral effects, yet these effects are generally modest and uneven across contexts. The fact that seven of 12 trials achieved statistically significant reductions in BMI or BMI z‐scores underscores the promise of early intervention, particularly considering that early weight trajectories tend to track into later childhood and adulthood [45]. Some of the more comprehensive programs also demonstrated improvements in secondary outcomes, such as diet quality, motor competence, and parental practices, suggesting that even when weight changes are small, the intervention can beneficially influence multiple domains of child health.

Moreover, the clinical relevance of small BMI reductions should not be underestimated: In young children, even slight stabilization of weight gain can alter fat accretion trajectories and reduce the risk of insulin resistance, hypertension, and dyslipidemia over time. For example, meta‐analytic evidence indicates that interventions combining diet and exercise can reduce central adiposity (waist circumference) with moderate effect sizes, an outcome more tightly linked with metabolic risk than weight alone [46]. In addition, the timing of intervention may matter: Intervening before adipocyte hypertrophy becomes entrenched may enable reversal of fat cell development, thereby amplifying long‐term metabolic benefit.

### 4.1. Intervention Characteristics and Effectiveness

In this review, interventions of longer duration (≥ 12 months) and greater intensity—including refreshers or booster sessions—consistently yielded more robust results. This aligns with findings from obesity prevention in older children, where sustained interventions over multiple years are more likely to produce stable effects [47]. Importantly, parental engagement emerges repeatedly as a critical lever: Trials incorporating structured parent education, home‐based tasks, or counseling reported larger effects than those confined to the preschool environment [48, 49]. This underscores that behavior change at the child level often depends on simultaneous changes in the home environment.

Cultural tailoring and contextual relevance further influenced outcomes. Interventions adapted to the cultural, socioeconomic, or linguistic context of the families tended to achieve better adherence and effect sizes [50]. Moreover, combining pedagogical strategies with structural changes (e.g., menu redesign, playground modifications, and staff training) resulted in additive benefits, compared to purely educational designs. Use of implementation science principles—such as pilot testing, fidelity monitoring, and feedback loops—can further amplify the effectiveness of complex interventions.

Another important consideration is measurement and outcome harmonization. The COS‐EPOCH initiative proposes a standard set of outcomes for early childhood obesity trials—covering anthropometry, diet, physical activity, sleep, and family environment [51]. Adoption of such consensus frameworks would reduce heterogeneity across future trials and facilitate meaningful meta‐analytic synthesis.

### 4.2. Implementation Fidelity and Process Evaluation

The evaluation of implementation fidelity and process evaluation was explicitly reported in eight of the included studies. This measurement is crucial because differences in outcomes, such as the mixed results observed in BMI z‐scores, may be due to the quality or extent of intervention delivery rather than the intervention’s core effectiveness. The methods used to assess implementation varied across the trials, falling into several common categories used in public health research.

A key set of methods involved quantifying participation and delivery. Several studies utilized Attendance and Participation Logs to track the dose of the intervention received. For instance, Nyberg et al. [38], Rhee et al. [34], and Gato‐Moreno et al. [35] evaluated participant attendance and satisfaction with sessions, noting that the variability in participation levels across groups may have contributed to the mixed results observed in BMI outcomes. Another widely used technique involved the use of Teacher/Staff Checklists and Reports. The CHILE study [33] used weekly staff‐completed forms to rigorously assess the dose delivered and the adherence to the curriculum, thereby providing evidence of robust implementation. Natale et al. [36], Reyes‐Morales et al. [37], and Zask et al. [43] similarly focused on measuring staff adherence to new nutrition and physical activity policies or assessing the use of materials within childcare centers and preschools.

Furthermore, some studies employed more direct quality assurance methods, such as Direct Observation. Fitzgibbon et al. [44] utilized fidelity checklists completed by independent observers to ensure that the “Hip‐Hop to Health Jr.” curriculum was delivered exactly as intended. The noted variability in implementation fidelity measured across these trials provides essential context for the observed anthropometric results. For example, lower parental engagement reported by Nyberg et al. [38] could account for the lack of a significant BMI effect, whereas studies with demonstrated high curricular adherence (e.g., Fitzgibbon et al. [44]) tended to report more favorable outcomes in overweight prevention, underscoring the necessity of robust implementation for achieving positive public health results.

### 4.3. Mechanistic Considerations

The beneficial effects of these interventions likely operate through multiple intertwined pathways. On the energy‐balance side, structured physical activity components increase expenditure, while dietary improvements reduce intake of energy‐dense foods. Enhanced motor competence resulting from early movement training may raise children’s self‐efficacy for activity, supporting sustained increases in movement over time.

Behavioral and cognitive mechanisms are equally pivotal: Several interventions included elements of self‐regulation, inhibitory control training, or limit‐setting on discretionary foods, which can strengthen executive function and reduce impulsive snacking [34]. Modeling by caregivers and teachers, combined with environmental cues (e.g., limiting unhealthy food visibility), supports normative behavior learning. Additionally, alterations in parental feeding practices and home food availability may mediate the intervention impact, as observed in trials showing reductions in unhealthy food exposure at home.

From a biological standpoint, modest reductions in adiposity during early childhood may influence adipocyte differentiation, inflammatory signaling, and metabolic programming. According to the Developmental Origins of Health and Disease (DOHaD) paradigm, early‐life environmental exposures can affect epigenetic regulation of metabolic genes, establishing longer‐term susceptibility or resilience [52]. While direct mechanistic studies in preschoolers remain limited, this theoretical framework points to the importance of intervening early, before epigenetic patterns become fixed.

Beyond these, psychosocial stress and early‐life adversity may modulate obesity risk via neuroendocrine pathways (e.g., cortisol dysregulation) and promote unhealthy eating or sedentary behaviors [53]. Interventions that reduce stress or promote self‐regulation may therefore have additional indirect benefits in mitigating obesity risk.

### 4.4. Public Health Implications

At the public health scale, embedding multifactorial obesity prevention programs in preschools presents an efficient and equitable approach, given high enrollment rates in early childhood education in many settings. Programs that integrate structural interventions (e.g., healthy menus and active play infrastructure), staff training, and built‐in parental involvement can generate broader reach and impact. The fact that interventions in disadvantaged or minority groups often yield appreciable benefits suggests a potential to reduce health inequalities [38, 42]. Aligning preschool programs with community‐level initiatives (e.g., municipal health policy and family support services) may enhance sustainability and scale.

Policymakers and funders should prioritize interventions grounded in evidence, cost‐effectiveness, and scalability. Although economic evaluations in preschool‐aged interventions are rare, recent analyses (e.g., in telephone/SMS interventions) reveal important socioeconomic gradients in cost‐effectiveness: In one study, the incremental cost‐effectiveness ratio (ICER) was far more favorable in lower socioeconomic position (SEP) groups [54]. These findings suggest that prioritizing resource allocation to underserved populations could maximize health equity. Program designs should consider cost containment strategies (e.g., remote delivery and digital reinforcement) without sacrificing effectiveness.

Hybrid implementation–effectiveness trials are needed to bridge the gap between research and practice, allowing real‐world adaptation across geographic, cultural, and socioeconomic contexts. Strategies, such as stepped‐wedge designs or pragmatic trials, can support translation into policy without sacrificing methodological rigor.

### 4.5. Limitations and Future Research Directions

This body of literature, though promising, suffers from several pervasive limitations. All included trials were categorized as having high risk of bias, primarily due to challenges in blinding participants or implementers, selective outcome reporting, and high attrition in some studies. Heterogeneity in intervention design, measures of adherence, and follow‐up duration precluded robust meta‐analysis and limited cross‐study comparability. Additionally, most trials had relatively short postintervention follow‐up, making it difficult to assess long‐term sustainability of effects across school years.

Future research should adopt rigorous, pragmatic trial designs that marry internal validity with external applicability. Cluster randomized, blinded (where feasible), and well‐powered studies with strategies to minimize dropout are essential. Long‐term follow‐up extending into later childhood and adolescence is critical to evaluate durability of effects and potential “catch‐up” in weight. Mechanistic substudies embedded in trials should explore mediation by behavior, cognition, home environment, and biological markers (e.g., inflammatory cytokines and epigenetic signatures). Cost‐effectiveness evaluation should be integrated from the outset, using modeling to project long‐term health and economic outcomes; recent models suggest that intervention cost‐effectiveness may differ by socioeconomic strata [54]. Further, hybrid implementation–effectiveness frameworks would allow adaptation across contexts, while fidelity and process evaluation can elucidate implementation barriers and facilitators. Investigating digital or remote reinforcement modalities (e.g., SMS and app‐based reminders) may enhance scalability. Finally, incorporation of stress‐reduction components or self‐regulation training might potentiate effects, particularly in socioeconomically vulnerable populations, by addressing psychosocial pathways contributing to obesity risk [53].

## 5. Conclusions

This systematic review provides comprehensive evidence that multifaceted preschool‐based interventions integrating nutrition and physical activity can exert favorable effects on obesity‐related outcomes, particularly in reducing BMI and BMI z‐scores, enhancing dietary behaviors, and improving motor competence. While the magnitude of anthropometric changes is often modest, these early improvements are clinically relevant given the strong tracking of childhood obesity into later life and its association with long‐term cardiometabolic risks. Importantly, interventions that combine educational components with structural modifications in the preschool environment, and those that actively engage families, tend to demonstrate the most consistent benefits. These findings reinforce the preschool years as a critical window for obesity prevention, where even small changes in weight trajectories may translate into substantial health gains across the lifespan.

Despite these encouraging results, the current body of literature highlights important methodological and translational challenges. Most trials present high risk of bias, short follow‐up, and heterogeneity in outcomes, limiting comparability and synthesis. Future research should therefore adopt rigorous trial designs with longer‐term follow‐up, standardized outcome reporting, and integration of cost‐effectiveness analyses. Equally, hybrid effectiveness–implementation frameworks are needed to ensure scalability and real‐world impact, particularly in disadvantaged populations where obesity prevalence and its sequelae are greatest. Ultimately, embedding well‐designed, culturally sensitive, and family‐centered interventions within preschool systems holds promise not only for curbing early obesity but also for fostering healthier trajectories of growth, behavior, and well‐being throughout life.

## Conflicts of Interest

The authors declare no conflicts of interest.

## Funding

The authors received no potential support for the research, authorship, and/or publication of this article.

## Data Availability

Data sharing is not applicable to this article as no datasets were generated or analyzed during this study.
